# Supplementary material for: Estimates and determinants of health facility delivery in the Birhan cohort in Ethiopia
Source: PLoS One. 2024 Jul 26;19(7):e0306581. doi: 10.1371/journal.pone.0306581 (PMC11280242; doi:10.1371/journal.pone.0306581)
Supplement: S1 Table — (DOCX) [file pone.0306581.s001.docx]

**S1 Table:** Multivariate poisson regression analysis with robust error variance for determinants of health facility delivery

Generalized linear models No. of obs = 2,383

Optimization : ML Residual df = 2,366

Scale parameter = 1

Deviance = 959.8033022 (1/df) Deviance = .405665

Pearson = 625.5466202 (1/df) Pearson = .2643899

Variance function: V(u) = u [Poisson]

Link function : g(u) = ln(u) [Log]

AIC = 1.883258

Log pseudolikelihood = -2226.901651 BIC = -17438.49

------------------------------------------------------------------------------

| Robust

location_b | IRR Std. Err. z P>|z| [95% Conf. Interval]

-------------+----------------------------------------------------------------

edu |

Primary | 1.081387 .0290955 2.91 0.004 1.025839 1.139944

Secondary.. | 1.066629 .0320298 2.15 0.032 1.005663 1.13129

|

hftime_3cat |

0.5 to 1 hr | .9175291 .0204495 -3.86 0.000 .8783115 .9584977

>1 hr | .7755361 .0302758 -6.51 0.000 .71841 .8372047

|

parity |

primiparous | 1.175546 .0261134 7.28 0.000 1.125463 1.227858

|

anc_1to4 |

1 | 1.234976 .0928906 2.81 0.005 1.065698 1.431143

2 | 1.301006 .0941266 3.64 0.000 1.129004 1.499212

3 | 1.398246 .0998693 4.69 0.000 1.215589 1.608349

4+ | 1.484592 .1035981 5.66 0.000 1.294817 1.702181

|

ethn_cat |

Oromo | 1.052982 .0653758 0.83 0.406 .9323374 1.189239

Other | 1.191395 .0846718 2.46 0.014 1.036481 1.369463

|

rel_cat |

Muslim | .8080905 .0307383 -5.60 0.000 .7500356 .870639

|

wealthi~5cat |

Poorer | .900563 .043485 -2.17 0.030 .8192427 .9899554

Middle | 1.049681 .0471333 1.08 0.280 .9612495 1.146247

Richer | 1.209917 .0498309 4.63 0.000 1.116089 1.311634

Richest | 1.206256 .0507523 4.46 0.000 1.110774 1.309945

|

_cons | .5390332 .0425153 -7.84 0.000 .4618261 .6291476

------------------------------------------------------------------------------

Note: _cons estimates baseline incidence rate.
